# Taxonomic and Functional Features of Surface to Deep‐Sea Prokaryotic Communities in the Eastern North Pacific Ocean

**DOI:** 10.1111/1758-2229.70170

**Published:** 2025-08-06

**Authors:** Daniele De Corte, Leon Dlugosch, Abhishek Srivastava, Meinhard Simon, Dennis A. Hansell, Sarah Bercovici, Monica Orellana

**Affiliations:** ^1^ Ocean Technology and Engineering (OTE), National Oceanography Centre Southampton UK; ^2^ Institute for Chemistry and Biology of the Marine Environment (ICBM), University of Oldenburg Oldenburg Germany; ^3^ Department of Limnology and Bio‐Oceanography University of Vienna Vienna Austria; ^4^ Research Institute of Wildlife Ecology, University of Veterinary Medicine Vienna Vienna Austria; ^5^ Department of Ocean Sciences University of Miami Miami Florida USA; ^6^ Ocean BioGeosciences (OBG), National Oceanography Centre Southampton UK; ^7^ University of Washington Seattle Washington USA; ^8^ Institute for Systems Biology Seattle Washington USA

**Keywords:** diversity, marine ecosystem, metabolic potential, prokaryotes

## Abstract

Biogeochemical cycles in the ocean are strongly influenced by microbial activity, which affects nutrient and organic matter cycling. These processes, influenced by factors such as temperature, salinity, density and inorganic nutrients, drive the vertical stratification of microbial communities, which subsequently influence the chemistry at different depth layers. Sequencing technology has expanded our understanding of oceanic prokaryotic communities' taxonomic and functional potential. However, there is limited information on how these communities vary across gradients. In this study, we conducted metagenomic analyses on samples from the eastern North Pacific, collected across a longitudinal transect around 45°N and throughout the entire water column. We assessed taxonomic and functional classification, focusing on the roles of prokaryotic communities in biogeochemical cycling. Our results revealed that the surface community was dominated by the SAR11 clade, followed by Flavobacterales and Rhodobacterales. The deep layers harboured a more diverse community, where Thaumarchaeota accounted for the most significant proportion. This clear taxonomic stratification led to variations in the communities' functional capabilities across different depth layers. Photosynthesis and heterotrophy dominated the surface layers, whereas the deeper layers exhibited a mix of metabolic features, allowing organisms to potentially utilise both inorganic and organic carbon sources.

## Graphical abstract

The North Pacific Ocean is characterised by a complex interplay of microbial metabolic pathways, where key processes such as carbon fixation (via the Calvin cycle, HP/HB cycle and Wood–Ljungdahl pathway), sulphur oxidation and phosphonate metabolism take place at different pelagic zones. These processes are driven by diverse microbial communities that adapt to varying environmental conditions, such as nutrient and organic matter availability.

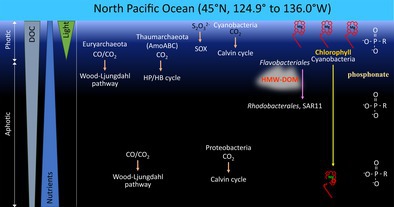

## 1 Introduction

Prokaryotes play a key role in the ocean's biogeochemical cycles by transforming organic matter and recycling nutrients (Azam and Malfatti 2007; Moran 2015). These oceanic crucial biological processes vary greatly across latitude (Dlugosch et al. 2022; Salazar et al. 2019) and pelagic zones (Deutschmann et al. 2024). Surface processes are dominated by photosynthesis, where inorganic carbon is transformed by phototrophs into organic carbon, making it available to heterotrophic organisms (Karl et al. 1998). The deep ocean mostly relies on surface‐derived carbon that sinks into the lower layers as particles and dissolved organic carbon transported via global thermohaline circulation (Arístegui et al. 2009; Herndl and Reinthaler 2013; Carlson et al. 2010).

Several studies have shown that the dark oceans' biogeochemical cycles are not solely dependent on the supply of organic carbon from the euphotic layers. Chemolithoautotrophy and mixotrophy also play critical roles in producing organic carbon through inorganic carbon fixation (Herndl and Reinthaler 2013; Nunoura et al. 2015; Swan et al. 2011). Therefore, the key biogeochemical pathways and players change with depth, following changes in controlling factors such as temperature, salinity, density, inorganic nutrients and organic matter. These factors exert strong selective pressures on microbial communities along the vertical water column, leading to a clear depth stratification of microbial communities in the open ocean (Xue et al. 2020; Liu et al. 2018).

Recent advances in sequencing technology have increased our understanding of the taxonomic and functional potential of prokaryotic communities. Although several studies have investigated the large‐scale distribution of prokaryotic communities in the ocean (Dlugosch et al. 2022; Salazar et al. 2019; Sunagawa et al. 2015), little information is available on how their taxonomic composition, functional traits and overall diversity vary across environmental gradients. To expand this information to the understudied temperate North Pacific, we collected samples for metagenomic analyses in the eastern North Pacific at 13 stations on a zonal transect around 45°N, ranging from coastal waters to the open ocean. Samples were collected throughout the water column, from the surface to the bathypelagic layers. The taxonomic diversity was assessed by extracting 16S rRNA gene fragments from the metagenomes (Logares et al. 2014), while the functional features were classified using KEGG orthologues (KOs). The main objectives of this study were (i) to taxonomically and functionally characterise the prokaryotic communities across the transect and different pelagic zones and (ii) to assess the factors controlling the shape of the prokaryotic communities, especially their functional capability.

## 2 Materials and Methods

### 2.1 Study Area and Sampling

Sampling was conducted on board the *R/V Oceanus* in August 2018. Water samples were collected at 13 stations through the entire water column along a zonal transect between 124.9°W and 136.0°W. Samples for biological (DNA sequencing) analyses were collected at four to six depths: the surface layer (2–20 m), the subsurface (25–100 m), the mesopelagic (200–450 m), the upper oxygen minimum zone (OMZ) (600 m) and the bathypelagic (2100–4145 m). Water samples were collected with 12‐L Niskin bottles mounted on a CTD (conductivity–temperature–depth) rosette sampler equipped with specific pressure, temperature, salinity, conductivity and dissolved oxygen sensors.

### 2.2 Inorganic Nutrient Concentrations

Samples for dissolved inorganic nutrients (nitrate, nitrite, orthophosphate and silicate) were measured by the Marine Chemistry Lab at the University of Washington, following the protocols of the WOCE Hydrographic Program using a Seal Analytical AA3 autoanalyzer (SEAL Analytical Inc., Mequon, WI, USA) (UNESCO 1994).

### 2.3 Dissolved Organic Carbon

DOC was quantified on a Shimadzu TOC‐VCSH total organic carbon analyser, equipped with an autosampler ASI‐V, via high‐temperature catalytic combustion in the Hansell Biogeochemistry Lab at the University of Miami as described in (Halewood et al. 2022). The analyses were quality controlled using a DOC deep‐sea reference material (Hansell Biogeochemistry Laboratory, University of Miami; Hansell 2005).

### 2.4 DNA Extraction and Sequencing

Depending on the depth, 1–5 L of water were filtered onto a 47‐mm membrane filter of 0.22 μm pore size (Millipore, GTTP) and stored at −80°C until further processing in the lab. DNA extraction was performed using the DNeasy Power Soil Pro Kit (QIAGEN) following the manufacturer's instructions. The extracted DNA's quality and concentration were assessed by Qubit dsDNA HS Assay Kit (Thermo Fisher Scientific). Illumina shotgun libraries were prepared by the Northwest Genomics Laboratory (University of Washington, WA, USA) using the Kapa Hyper library construction kit (Roche). Sequencing was performed by Illumina NovaSeq 6000 (Illumina Inc., San Diego, USA). The obtained reads were deposited in the NCBI Sequence Read Archive (SRA) under the BioProject accession number PRJNA1002880.

## 3 Bioinformatics Analyses

### 3.1 Metagenomic Assembly and Gene Prediction

Reads were quality checked, and adapter sequences were trimmed using Trimmomatic 0.36 (ADAPTER:2:30:10 SLIDINGWINDOW:4:25 MINLEN:100) (Bolger et al. 2014). The high‐quality reads were assembled using metaSPAdes 3.11 (Nurk et al. 2017). Coding sequences of the obtained contigs were predicted using Prodigal 2.6.2 (Hyatt et al. 2010). Genes shorter than 210 bp and longer than 4500 bp were discarded. Additionally, the sequences were clustered at 95% identity to generate a gene catalogue using USEARCH v10 (cluster_fast–id 0.95) (Edgar 2010). The obtained gene sequences were taxonomically classified using Kaiju 1.6 (Menzel et al. 2016) (greedy mode with five allowed substitutions and *e*‐value 10e^−5^) with the Refseq nr (O'Leary et al. 2015) and ProGenomes (Mende et al. 2017) databases and functionally assigned by using the Kyoto Encyclopedia of Genes and Genomes (KEGG) online annotation tool GhostKOALA (Kanehisa et al. 2016) (https://www.kegg.jp/ghostkoala/) using the KEGG gene database (release 86) with default settings.

### 3.2 Read Abundance

Reads longer than 75 bp were mapped to the obtained sequences using bowtie 2.3.5 (very‐sensitive‐local mode) (Langmead and Salzberg 2012). SAMtools version 1.9‐58‐gbd1a409 (Li et al. 2009) was used to produce the read abundance table from the alignment file. The obtained data was normalised by dividing read counts by the length of each gene in kb to obtain reads per kilobase (RPK). Subsequently, the sum of RPKs for each sample was divided by 10^6^ to obtain reads per kilobase per million (RPKM).

### 3.3 Metagenomics 16S rRNA Gene Tags

16S ribosomal RNA gene fragments were extracted from the metagenome reads as described by Logares et al. (2014). The extracted 16S miTags were further processed using USEARCH v11 (Edgar 2013, 2018). Briefly, the obtained miTags were clustered at 97% sequence identity, and a counts table was constructed by mapping the 16S reads to the final OTUs. Finally, the OTUs were taxonomically assigned using SINTAX (Edgar 2016) with the RDP 16S training set v16 database (Maidak et al. 2001). OTUs reported in more than 80% of the samples with detection of at least 0.1% were considered core phylotypes.

### 3.4 Data Processing

**FIGURE 1:**
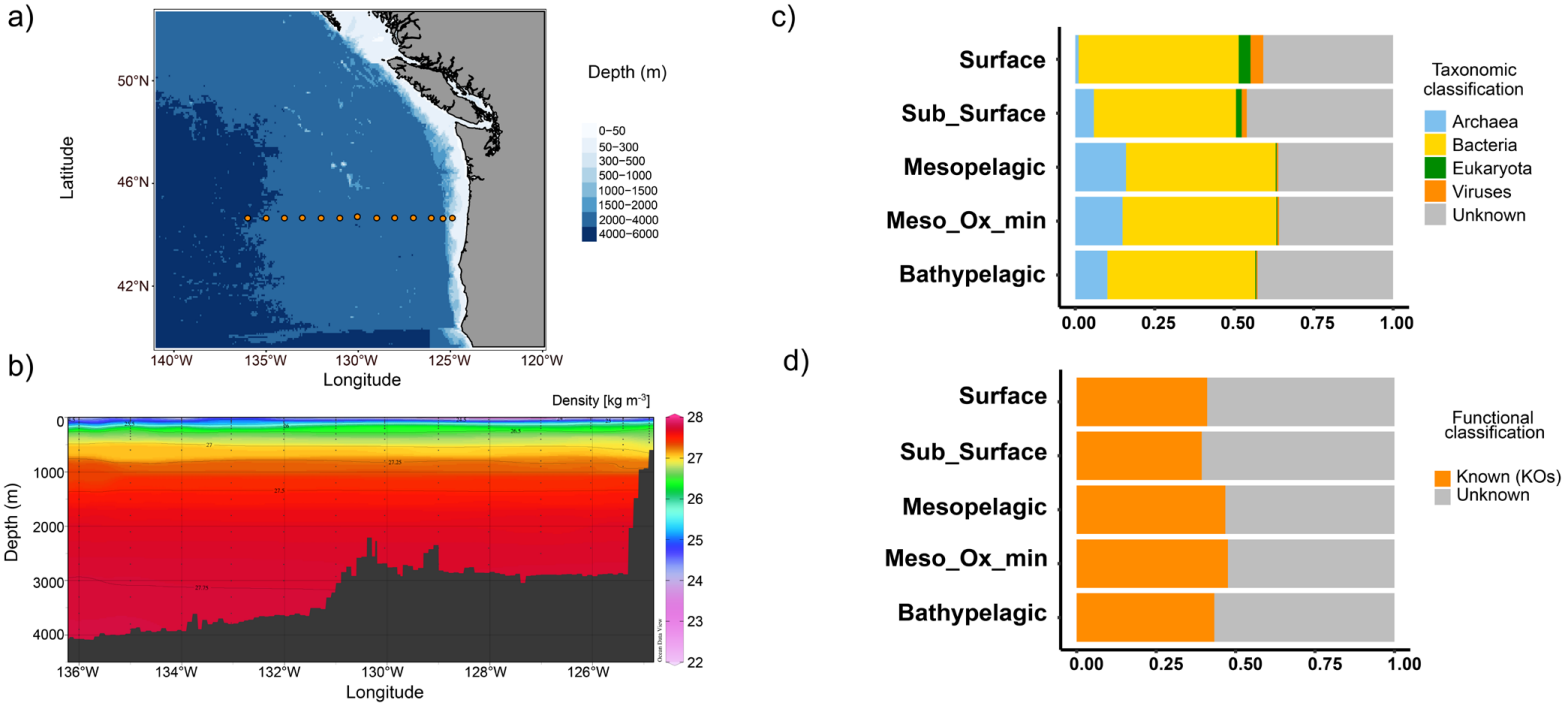
(a) Map of the station visited during the cruise in 2018, (b) density plot of whole depth profile and (c) percentages of taxonomic (Bacteria, Viruses, Archaea, Eukaryota and unclassified) and functional (KEGG and unclassified) annotations of gene sequences of the metagenomes.

All data processing and statistical analyses were conducted in R software v.3.6 (version 3.6.0; https://www.r‐project.org/) with the following additional packages: vegan (Oksanen et al. 2018), pheatmap (Kolde 2019), DESeq2 (Love et al. 2014), phyloseq (McMurdie and Holmes 2013), microbiome (Lahti and Shetty 2019) and ggOceanMaps (Vihtakari 2024). Linear interpolation was used to estimate missing nutrient values (*n* = 1) based on known values within the data set. Ocean Data View (ODV) (Schlitzer 2023) was used to calculate oxygen saturation and generate the density plot shown in Figure 1.

## 4 Results

### 4.1 Study Area Physicochemical Characteristics

There was a surface‐to‐depth stratification of water masses, with density values decreasing from a minimum of 22.5 at the surface to a maximum of 27.8 kg m^−3^ in the bathypelagic layers. Dissolved oxygen concentrations decreased with depth from the highest values of 271.9–291.9 μmol kg^−1^ (oxygen saturation 99.3%–105.7%) in surface and subsurface waters to 14.3–28.8 μmol kg^−1^ (oxygen saturation 4.6%–9.1%) at 600 m depth (Table 1). The oxygen concentrations below the mesopelagic oxygen minimum (OMZ) (600 m) increased towards the bathypelagic layers, ranging between 60.7 and 121.6 μmol kg^−1^ (oxygen saturation 18.1%–35.7%). Nitrate and phosphate concentrations increased with depth, reaching a maximum within the OMZ (43.5 and 3.19 μmol kg^−1^, respectively). Nitrite concentrations showed no trend among different depth layers (Table 1). Silicate increased with depth, with the lowest values detected at the surface (1.0 μmol kg^−1^) and the highest (162.7 μmol kg^−1^) in the bathypelagic layers. On the other hand, DOC showed an opposite trend, with its value declining from 78.1 at the surface to 36.4 μmol kg^−1^ in the bathypelagic layers (Table 1).

**TABLE 1:** Physico chemical characteristics of the sampled waters in the Eastern Nord Pacific.

	No. of samples	Depth	Temperature	Salinity	Density	Oxygen	Oxygen Sat	Si	NO_3_^−^	NO_2_^−^	PO_4_^3−^	DOC
		(m)	(°C)		(kg m^−3^)	(μmol kg^−1^)	(%)	(μmol kg^−1^)	(μmol kg^−1^)	(μmol kg^−1^)	(μmol kg^−1^)	(μmol kg^−1^)
Surface	15	2–20	10.5–18.5	31.5–32.7	22.5–24.9	227.2–271.9	95.8–99.3	1.0–9.6	0.03–1.99	0.00–0.10	0.10–0.57	59.3–78.1
Subsurface	22	25–100	7.5–14.8	32.4–33.7	24.2–26.1	132.7–291.9	46.6–105.7	1.7–28.0	0.09–25.13	0.01–0.54	0.39 1.82	48.2–67.4
Mesopelagic	5	200–450	7.8–5.7	33.9–34.1	26.5–26.9	43.7–147.2	14.3–50.5	37.9–64.8	28.25–39.14	0.01–0.03	1.91–2.76	41.9–52.2
Meso Ox‐min	11	600	4.2–5.0	34.1–34.2	27.0–27.1	14.3–28.8	4.6–9.1	51.7–91.2	22.9–43.5	0.00–0.02	2.06–3.19	39.6–48.8
Bathypelagic	22	2100–4145	1.5–1.9	34.6–34.7	27.7–27.8	60.7–121.6	18.1–35.7	92.7–162.7	21.6–42.5	0.00–0.09	1.87–2.95	36.4–46.8

### 4.2 Microbial Community Structure

**FIGURE 2:**
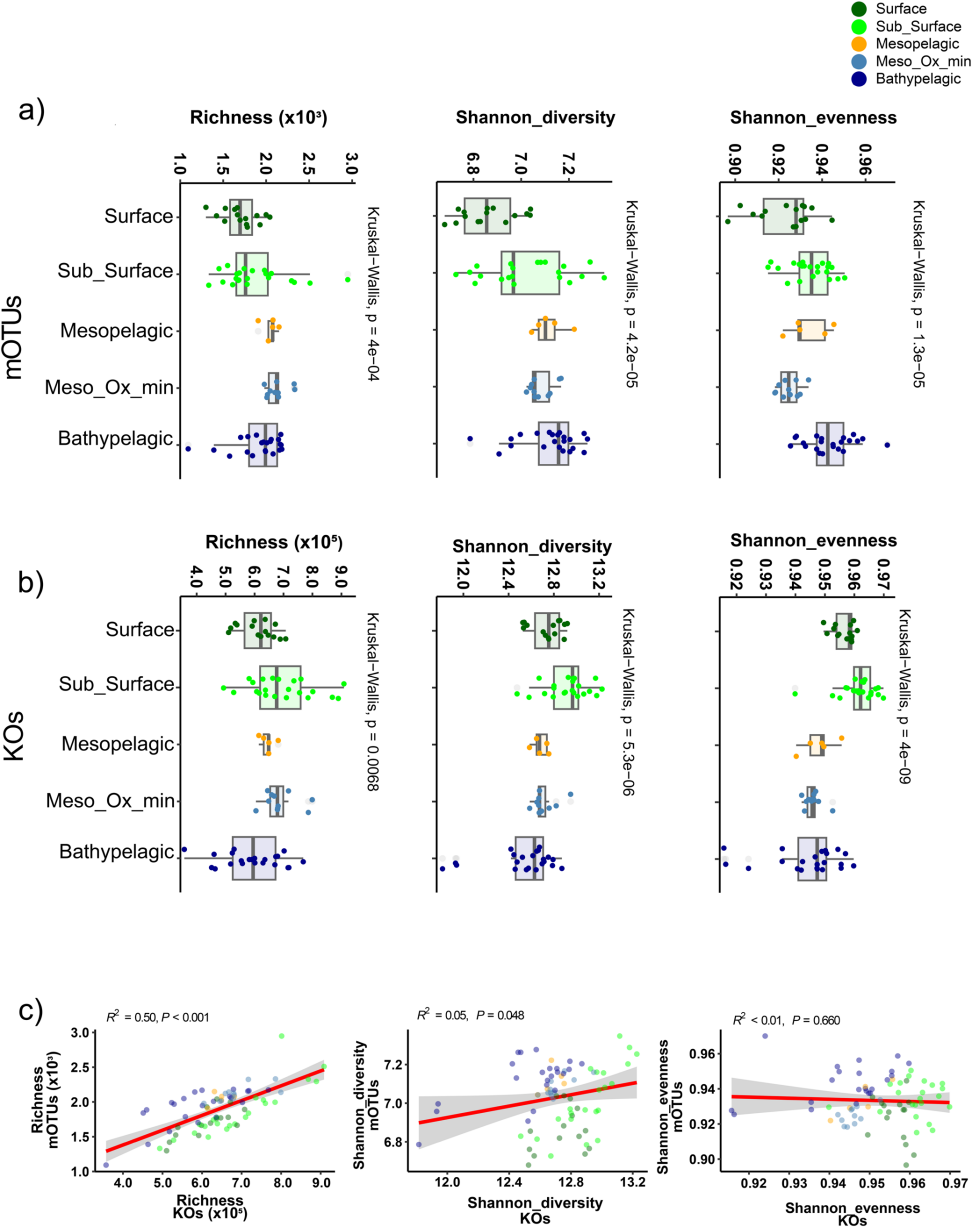
Richness, Diversity and evenness indexes of mOTUs (a) and KOs (b) profiles across five different pelagic zones. Relation between the mOTUs and KOs indexes (c).

**FIGURE 3:**
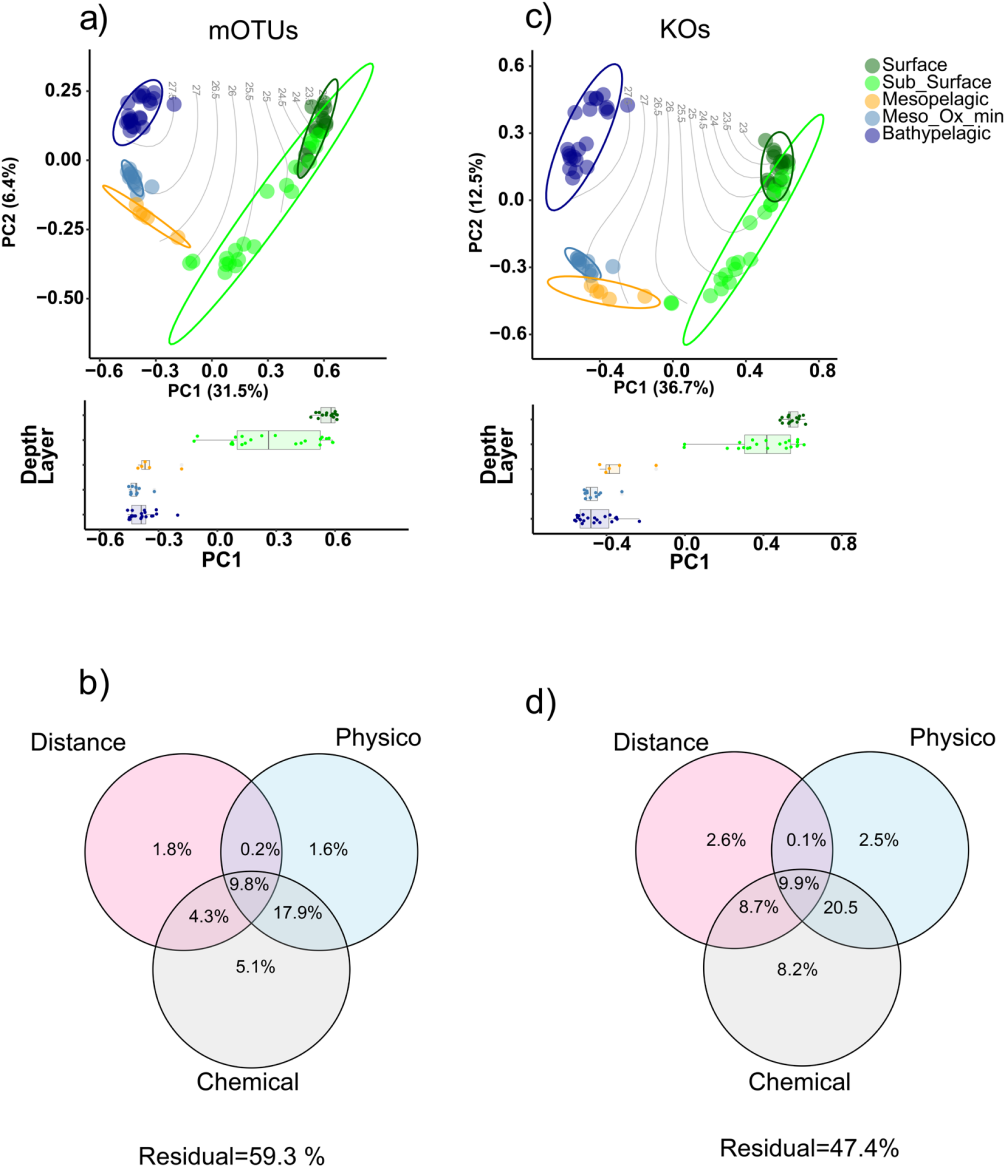
Principal component analyses (PCAs) of mOTUs (a) and KOs (c) profiles. Variation partitioning of physico‐chemical and spatial factors explaining the prokaryotic community composition of the taxonomic (b) and functional (d) profiles.

After assembly, a total of 6.3 × 10^6^ unique nr protein‐coding genes were predicted from the microbial community. About 44% of these coding genes were functionally annotated by homology to KEGG ORTHOLOGY (KO) (Figure 1d). The largest proportion of genes was affiliated with Bacteria (~47%), followed by Archaea (~10%), Eukaryotes (~1%) and Viruses (~1%) (Figure 1c). The Archaea contribution increased with depth and reached its maximum value in the mesopelagic layers, while the contribution of eukaryotes and viruses to the total microbial community declined with depth (Figure 1c). The 16S rRNA gene OTUs' taxonomic and KOs profiles varied significantly with depth in richness, diversity and evenness (Figure 2). Taxonomic and KOs richness were correlated (*R* ^2^ = 0.50, *p* < 0.001), while diversity and evenness showed different patterns between the two profiles. The taxonomic profiles displayed increased diversity and evenness from the surface to the bathypelagic layers, whereas those of the KOs profiles decreased. This resulted in a minimal relation between the two profiles in terms of Shannon diversity and evenness (Figure 2). The PCA of the metagenome extracted OTUs (mOTUs) and KOs profiles showed that the microbial communities were stratified following the changes in water density. PC1 and PC2 accounted for 31.5% and 6.4% of the variation in the mOTUs profiles, while the KOs PC1 and PC2 accounted for 36.7% and 12.5%, respectively. Variation partitioning showed that sample distance and physicochemical variables explained ~40% of the variation of the mOTUs profile and ~50% of the KOs profile, with physico‐chemical variables accounting for most of the variation in both profiles (Figure 3b,d).

**FIGURE 4:**
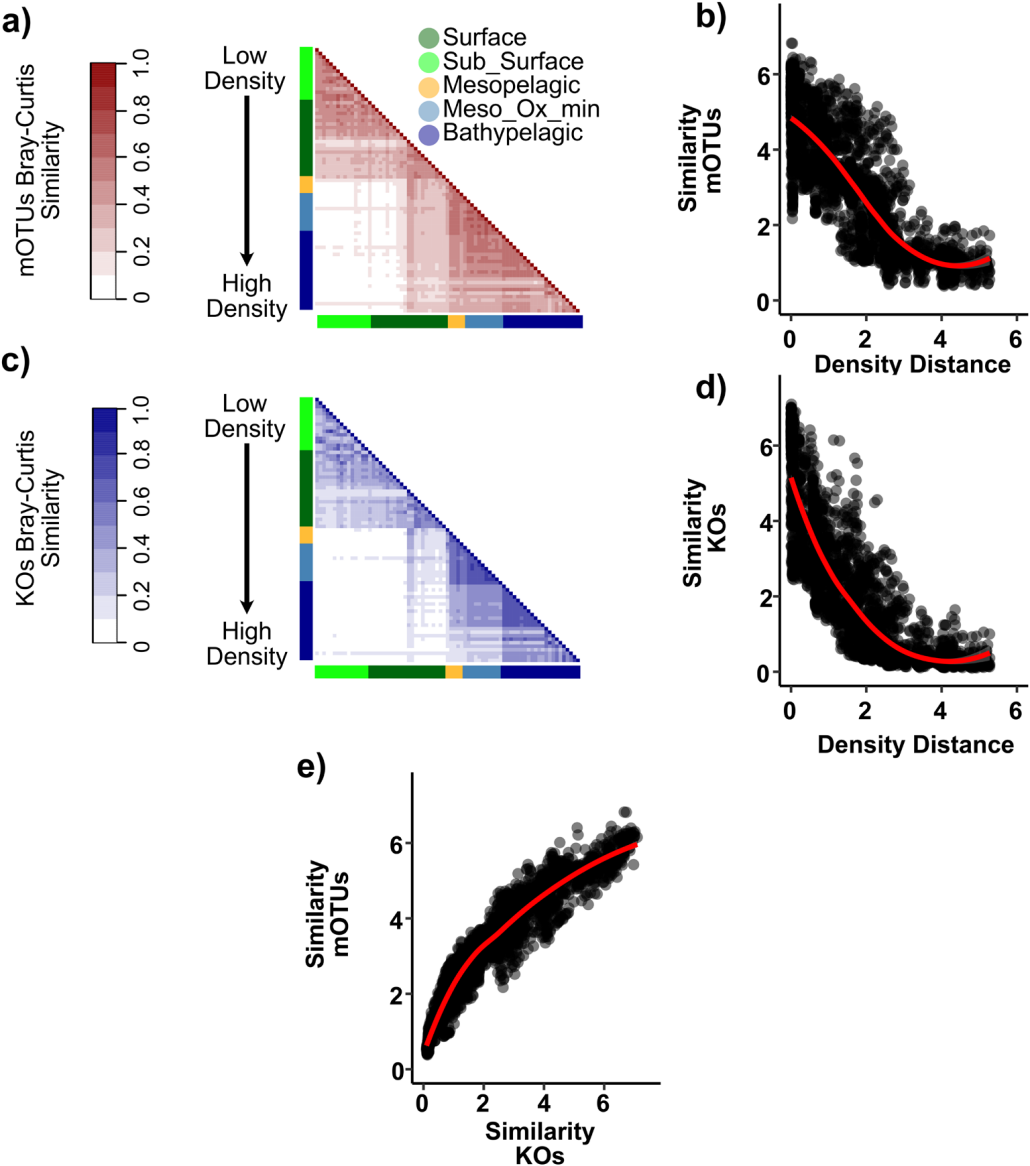
Similarity matrices of mOTUs (a) and KOs (c) profiles and their changes in comparison with density (b, d). Relation between the mOTUs and KOs Bray–Curtis similarities (e).

The taxonomic and functional profiles were grouped along the water column following the depth and density gradient as shown by the Bray–Curtis similarity matrices (Figure 4a,c). The relationships between density distance and the similarities of the microbial community of the mOTUs and KOs showed similar distribution, with the similarity of the microbial community declining with the increase in density differences. Samples collected at the same density (i.e., where the density distance is 0) showed a microbial similarity ranging between 20% and 70%, suggesting high microbial community variability within the same water mass. On the other hand, samples with high‐density distance showed very low similarity in microbial community composition, especially in the KOs profile (Figure 4b,d). Additionally, the KOs and mOTUs similarity profiles were highly correlated (*R* ^2^ = 0.88; *p* < 0.001; Figure 4e).

### 4.3 Taxonomic Structure of the Microbial Communities

**FIGURE 5:**
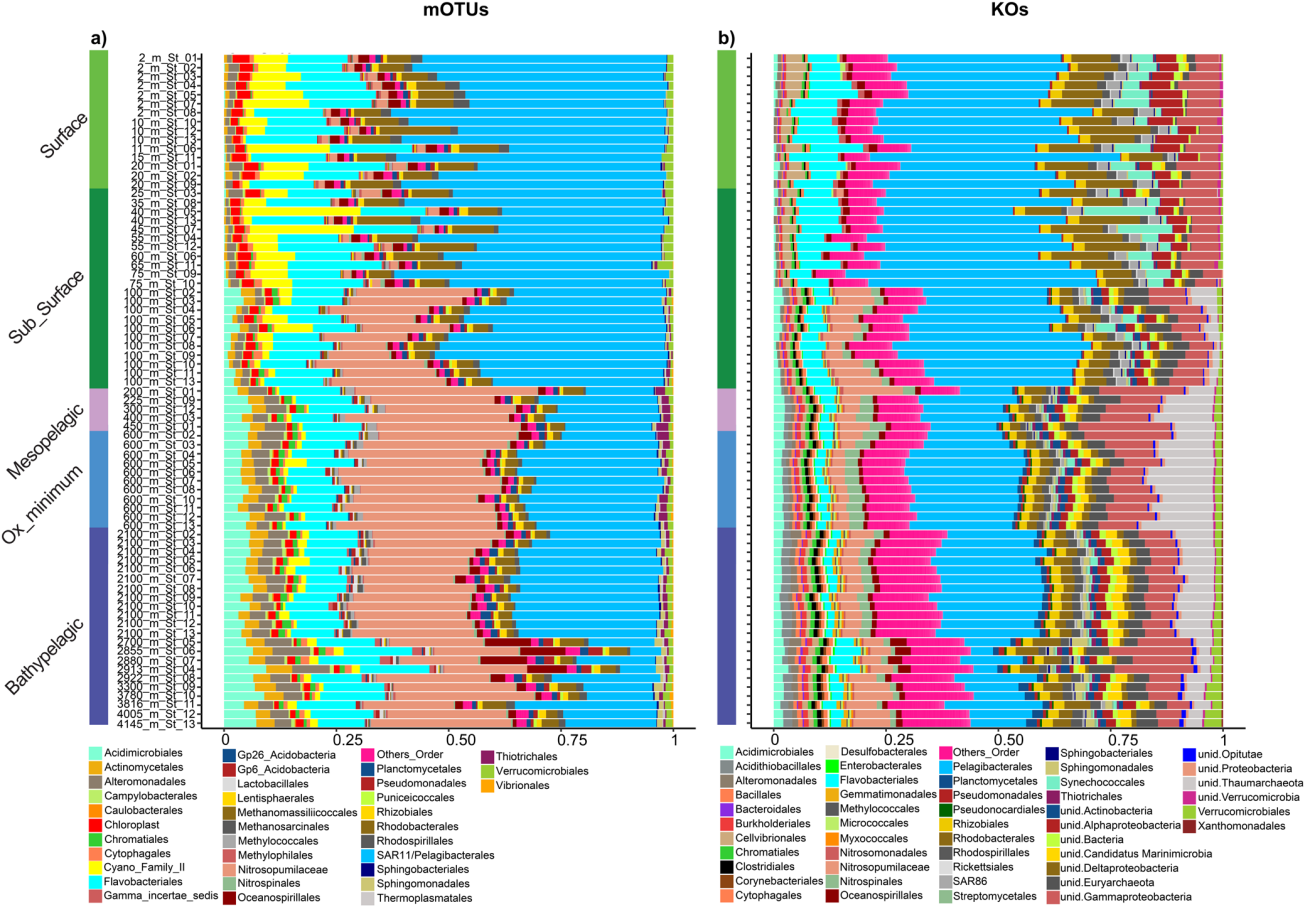
Taxonomic classification of the mOTUs (a) and KOs (b) profiles across the longitudinal transect and the water column.

Phylogenetic analyses of the extracted 16S rRNA genes (mOTUs) and the metagenome gene sequences (KOs) revealed the dominance of *Proteobacteria*, *Bacteroidetes*, *Thaumarchaeota* and *Actinobacteria* (Figure 5a,b). The microbial communities showed high taxonomic variability in both profiles, with clear changes in depth, with the KOs profiles showing higher resolution in the number of taxa found than the mOTUs. In contrast, microbial communities at the surface did not change from the near shore to the open ocean. The *Pelagibacterales* largely contributed to the microbial communities in both profiles; their contribution was larger in surface (reaching 33% in the KOs and 50% in the mOTUs profiles) and declined with depth, reaching their lowest values in the bathypelagic layers: ~15% in the KOs profiles and 24% in the mOTUs. *Flavobacterales* and *Rhodobacterales* also significantly contributed to the microbial communities in both profiles in the surface and subsurface layers. Their contribution declined with depth and reached lower values in the bathypelagic layers. In the mOTUs profile, *Nitrosopumilaceae* had the largest contribution in the mesopelagic and bathypelagic layers, reaching ~30% of the total communities. In the KOs profiles, unidentified Euryarchaeota and Thaumarchaeota also largely contributed to the meso‐ and bathypelagic community (Figure 5a,b).

In the taxonomic profile, 39 OTUs were assigned to *Candidatus* Pelagibacter, and 4 were putatively assigned to Unclassified Alphaproteobacteria, which composed the core surface microbial community. Ninety‐one taxa formed the mesopelagic core community. The most abundant taxa of the mesopelagic core community were assigned to *Candidatus* Pelagibacter (18%), *Nitrosopumilus* (16%), followed by unassigned Bacteria (16%), Proteobacteria (14%), Gammaproteobacteria (14%) and Thaumarchaeota (10%). The bathypelagic core community was composed of 53 taxa dominated by mOTUs with similar taxonomic affiliations as those found in the mesopelagic core community; ~92% of the bathypelagic core community was shared with the mesopelagic community. All three depth layers shared nine core taxa identified as *Candidatus* Pelagibacter (Figure S1).

Among the potential nitrifiers, apart from *Nitrosopumilaceae* that dominated the meso‐ and bathypelagic zones, *Nitrosomonadales* (which includes five genera of ammonia‐oxidising bacteria) were also found in the surface (~1%) layers. (*Nitrosomonadales* abundance is only displayed in the KOs profile as in the mOTUs profile their median across the samples was below 0.1%). Nitrite‐oxidising bacteria associated with the *Nitrospinales* order were also detected in both profiles, with their contribution maximised in the OMZ and mesopelagic layers.

### 4.4 Functional Analysis of Microbial Community

**FIGURE 6:**
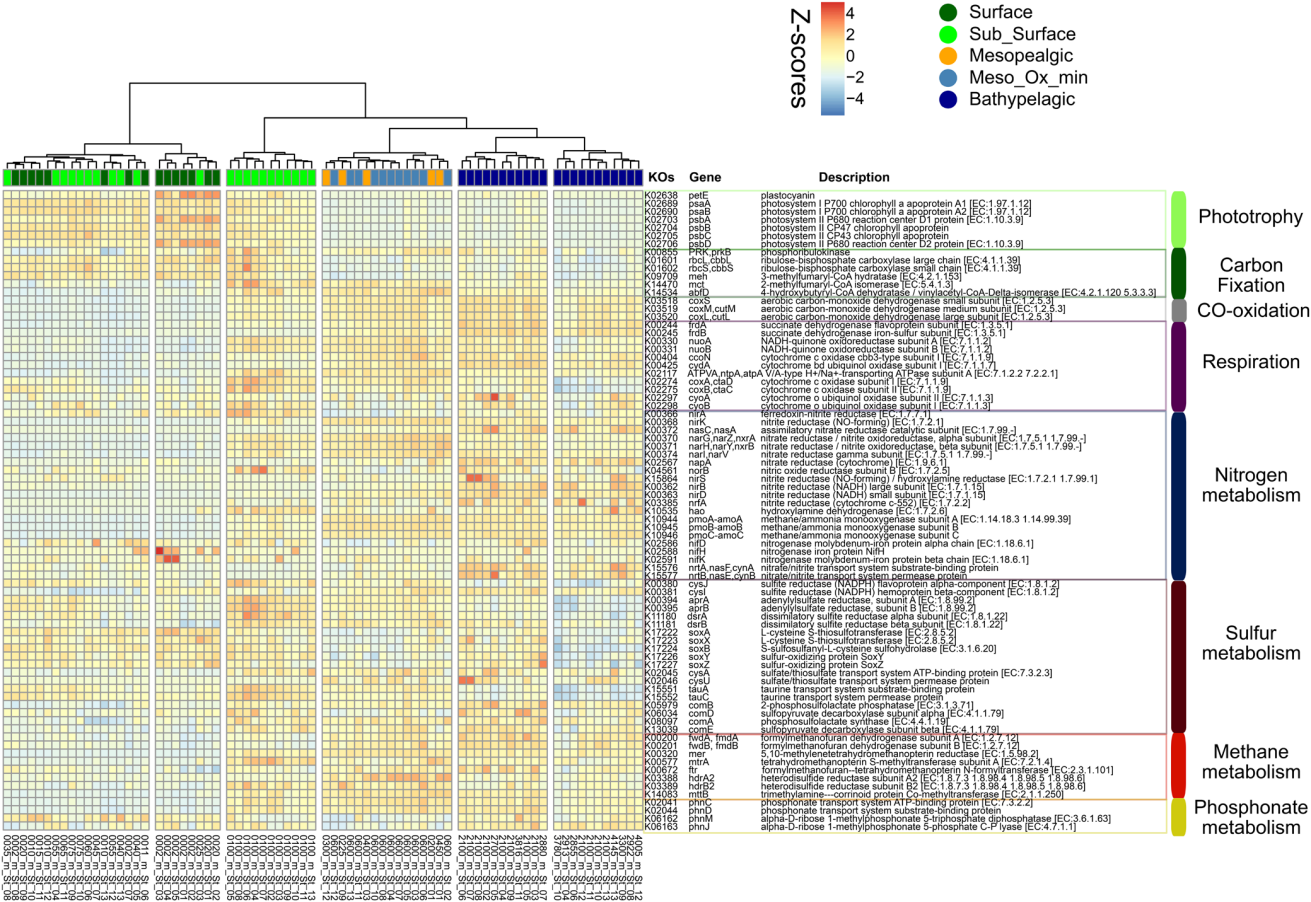
Heatmap of selected genes (KOs) accounting for phototrophy, carbon fixation, CO‐oxidation, respiration, nitrogen, sulphur, methane, and phosphonate pathways across the longitudinal transect and the water column.

**FIGURE 7:**
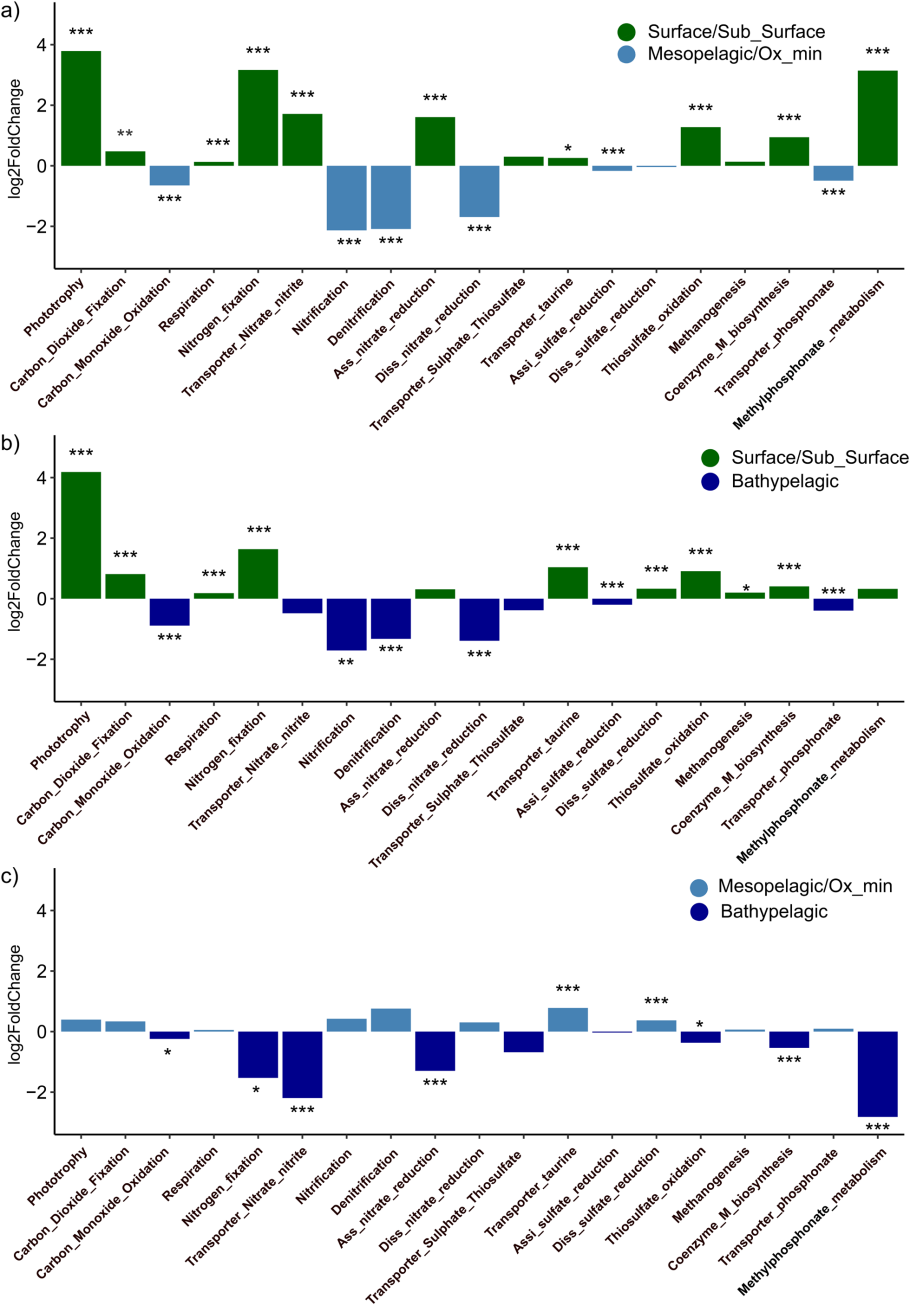
Changes of the selected metabolic pathways between surface/subsurface, mesopelagic/oxygen‐minimum and bathypelagic layers. (***p < 0.001; **p < 0.01; *p < 0.05).

The metagenomic data obtained from the microbial communities were used to characterise potential metabolic pathways. The KOs showed a clear depth‐dependent pattern, with most assigned genes clustering within different depth layers (Figure S2). To explore the potential metabolic differences between microbial communities inhabiting different pelagic zones, marker genes related to relevant pathways in the open ocean were selected (Figures 6 and 7). These marker genes were characterised by their functions in phototrophy, carbon fixation, CO‐oxidation, respiration, nitrogen, sulphur and methane‐phosphonate metabolisms.

The surface community was dominated by phototrophic mechanisms, along with genes encoding for carbon dioxide and nitrogen fixation. Carbon monoxide oxidation genes were significantly higher in the meso‐ and bathypelagic layers compared to the surface ocean (Figures 6 and 7). This process involves converting carbon monoxide into CO_2_, using oxygen as the primary electron acceptor, or alternative acceptors like nitrate or sulphate in low‐oxygen conditions (King and Weber 2007). Other processes that occur where oxygen is limited or absent, such as denitrification and dissimilatory nitrate reduction, were up to twofold higher in the meso‐ and bathypelagic layers than in the surface ocean. On the other hand, assimilatory nitrate reduction, a process that converts nitrate (NO_3_^−^) to ammonium (NH_4_^+^), which is then incorporated into organic molecules, dominated in the epipelagic layers. This process is particularly important for the uptake of organic nitrogen by primary producers (both eukaryotes and cyanobacteria).

While genes associated with sulphur metabolism did not show clear differences between depth layers, thiosulfate oxidation was primarily found in the surface ocean (Figures 6 and 7). Genes associated with methanogenesis did not show a clear trend through the water column. In contrast, coenzyme M biosynthesis, another gene involved in the methane cycle, was predominant in the surface ocean. Methylphosphonate metabolism genes, involved in the degradation of organic matter that serves as a source of phosphorus, had a higher contribution in the surface and bathypelagic layers. In contrast, their contribution was minimal in the mesopelagic layers (Figures 6 and 7).

## 5 Discussion

### 5.1 Factors Driving Prokaryotic Taxonomic and Functional Composition in the Water Column

Understanding the factors that shape prokaryotic taxonomic and functional composition throughout the water column is essential for interpreting microbial biogeography and ecosystem functioning. Surface‐to‐deep ocean profiles across a transect provide important insights into vertical and regional patterns. Such comprehensive data sets are vital for achieving broader spatial coverage and uncovering region‐specific drivers of microbial community structure and function.

The mOTUs' richness and diversity increased from surface to the meso‐ and bathypelagic layers, while the KOs indexes appeared lower in the deeper layer, suggesting a reduced functionality of the prokaryotic community in the dark ocean within a broader community. The dark ocean harbours a wide range of taxonomically diverse organisms that survive under limited carbon sources (Arrieta et al. 2015). In fact, mOTU diversity had a negative relationship with DOC (*R* = −0.61, *p* < 0.001) (Figure S3), suggesting that the microbial community increases its diversity as the concentration of available substrate (measured here as DOC) decreases. Both chemical recalcitrance of individual molecules and their individual diluted concentrations play a role in limiting microbial utilisation of DOC as a substrate (Arrieta et al. 2015; Bercovici et al. 2021; Shen and Benner 2018).

Deep‐sea microbes rely primarily on labile dissolved organic matter derived from sinking particles from surface waters as a source of growth (Arístegui et al. 2009; Bercovici et al. 2021; Moran et al. 2016; Hansman et al. 2009). While bacterial diversity (Shannon diversity) is high at depth, there are localised areas where it decreases at the seafloor and reflects the Shannon diversity values in the surface waters; this could be due to the solubilisation of sinking particles or resuspension of sediment organic matter (Figure S4b,c).

The surface ocean appears to have different controls on its microbial community. Where the surface DOC concentration is highest, mOTU diversity is highest on the surface (upper ~100 m; Figure S4). This high concentration of available substrates in the surface ocean increases microbial diversity and provides more opportunities for a wider variety of microbial communities to grow.

The functional similarity of the prokaryotic community in waters with identical density was higher than taxonomic similarity (Figure 4). This outcome suggests that substrate composition may be selected by different communities that occupy the same ecological niche, indicating that different taxa perform the same ecological function. This redundancy in ecological roles suggests that the communities encode for similar metabolic genes, reflecting redundancy in metabolic capability. Under limiting and stable environmental conditions, closely related species may occupy different ecological niches. While this can lead to high taxonomic diversity, it may not translate into increased functional diversity if specific groups perform similar ecological roles despite their taxonomic differences. Although there may also be adaptation differences among genes associated with a single KO due to varying environmental conditions, this may suggest high niche partitioning among gene variants, as demonstrated by Dlugosch et al. (2022). Our results support the notion that environmental conditions play a key role in shaping the distribution of functional groups while also affecting the taxonomic composition (Louca et al. 2016).

The taxonomic and functional profiles revealed clear differences in bacterial communities across depth (Figures 3 and 4). These results showed that vertical stratification driven by changes in physico‐chemical variables over the different depth layers is a key factor in shaping bacterial community structure in the water column. Our correlation analysis provides new insights by highlighting specific associations between environmental variables and community composition, contributing to a more detailed understanding of these relationships. Subsurface (20–100 m) layers exhibited high richness and diversity in both profiles, indicating that this zone is a hotspot for microbial activity driven by environmental factors and resource availability (Figure 2). This zone, which includes the deep chlorophyll maximum, is characterised by high phytoplankton biomass and production (Cullen 2015), which can promote complex microbial interactions that influence prokaryotic productivity and diversity (Sunagawa et al. 2015; Marañón et al. 2021; Hu et al. 2022).

### 5.2 Taxonomic Profiling of the Prokaryotic Community

The taxonomic classification of the prokaryotic community along the transect and through the entire water column was conducted using metagenome‐extracted 16S rRNA gene (mOTUs) and gene data (KOs). In general, the two profiles were comparable. Nevertheless, the KOs data showed higher taxonomic resolution than the 16S rRNA gene sequences at all taxonomic levels, as previously found in different studies (Durazzi et al. 2021; Khachatryan et al. 2020). Specific orders of prokaryotes showed different distribution patterns across various depths (Figure 6). The SAR11 clade, as previously described (Vergin et al. 2013; Salter et al. 2015; Ortmann and Santos 2016; Morris et al. 2002; Milke et al. 2022), dominated the surface and subsurface prokaryotic community in both profiles, with mOTUs taxonomically assigned as *Candidatus* Pelagibacter that constituted 90% of the core surface community. The physiological adaptability traits (such as efficient energy acquisition strategies) of members of the SAR11 clade make this group one of the most abundant members of the open water prokaryotic community that greatly contribute to the ocean's biogeochemical cycles (Giovannoni 2017). Nevertheless, its contribution was not only limited to the surface ocean but also represents a large proportion of the prokaryotic community in the meso‐ and bathypelagic layers. Several SAR11 genotypes are specialised for living under specific environmental conditions (López‐Pérez et al. 2020). A deep SAR11 ecotype, which contains genomic adaptations to the deep ocean and is phylogenetically distinct from the surface clades, was discovered in mesopelagic waters (Thrash et al. 2014). These findings suggest that different SAR11 types occupy different ecological niches in different pelagic zones.

*Flavobacterales* and *Rhodobacterales* also contributed to the surface and subsurface communities, and their abundance peaked where the DOC concentration was higher (Figure S4). These groups play an important role in phytoplankton–prokaryotic interactions by metabolising organic matter of phytoplankton origin (Buchan et al. 2014; Luria et al. 2017). The hydrolytic activity of *Flavobacterales* on high molecular weight DOM and in particular polysaccharides (Reintjes et al. 2019) may promote the release of low molecular weight organic matter that can be used by other members of the prokaryotic community, such as *Rhodobacterales* and SAR11.

Orders associated with Archaea showed very low abundance in the euphotic layers. Their contribution significantly increased and reached its largest proportion in the meso‐ and bathypelagic layers, where they were also a main member of the core prokaryotic community together with other groups. The lower abundance in the upper euphotic zone is consistent with previous studies showing the high sensitivity of ammonia‐oxidising Archaea to photoinhibition (Nunoura et al. 2015; Merbt et al. 2012). Many marine Thaumarchaeota, such as *Nitrosopumilus*, are involved in the oxidation of ammonia, a crucial step in the nitrogen cycle. This metabolic capability allows them to exploit a niche where they can use ammonia as an alternative source of energy when bioavailable organic matter is limited, as it often is in the meso‐ and bathypelagic ocean (Wright and Lehtovirta‐Morley 2023; Sintes et al. 2013). Ammonia‐oxidising Bacteria, such as *Nitrosomonas* and *Nitrosospira*, were predominantly found in the surface layers, indicating a niche separation between ammonia‐oxidising Bacteria and Archaea in different pelagic zones (Nunoura et al. 2015).

Additionally, the co‐occurrence of ammonia‐oxidising Archaea and nitrite‐oxidising Bacteria below the euphotic layers suggests a complementary relationship in the nitrification process, where the spatial and functional niche differentiation across various pelagic zones ensures an efficient nitrogen cycle in which each group specialises in different stages of the process.

### 5.3 Functional Characterisation of the Prokaryotic Community

Chlorophyll‐subunit‐encoding genes were primarily detected in the surface and sub‐surface ocean layers. However, they were also present in lower abundance in deeper layers, suggesting that sinking particles of photosynthetic origin are actively transported to the ocean's interior and may serve as additional bioavailable organic carbon (through particle solubilisation) for deep heterotrophic marine microbes (Dang et al. 2024; Agusti et al. 2015; Nagata et al. 2000; Hansell and Ducklow 2003). In our study, carbon fixation was associated with diverse microbial taxa. Sunlight‐driven primary productivity was linked to ribulose‐1,5‐bisphosphate carboxylase (RuBisCo) subunit genes. Interestingly, genes encoding both the small and large subunits of RuBisCo were also found in the dark ocean (Figure 6), in agreement with earlier studies that reported RuBisCo proteins (Orellana and Hansell 2012) and high rates of inorganic carbon fixation in bathypelagic waters (Reinthaler et al. 2010). These findings suggest that carbon fixation via RuBisCo is not limited to the surface ocean but is a widespread process that may also occur via chemolithoautotrophy in the dark (Swan et al. 2011; De Corte et al. 2021). It is also possible that a portion of the RuBisCo‐related genes detected in deep waters originate from phytoplankton transported by sinking particles from the photic zone (Orellana and Hansell 2012). The mesopelagic layers also contained genes for the 3‐Hydroxypropionate/4‐Hydroxybutyrate (HP/HB) cycle, specifically encoding 4‐hydroxybutyryl‐CoA dehydratase (4HBD), which were predominantly linked to Thaumarchaeota. The presence of ammonia monooxygenase‐encoding genes (*amo*A, *amo*B and *amo*C) in the mesopelagic zone further suggests a link between the HP/HB cycle and carbon fixation in archaeal members of the *Nitrosopumilaceae* family, as previously reported (Könneke et al. 2014). Carbon monoxide dehydrogenase (CODH), encoded by the genes *cox*L, *cox*M and *cox*S, mediates the aerobic oxidation of CO. The increased abundance of these genes in the mesopelagic and bathypelagic layers is consistent with earlier findings (Martín‐Cuadrado et al. 2007) and suggests that deep‐sea microbes may oxidise carbon monoxide as an alternative energy source in environments where energy and bioavailable organic matter are scarce or only sporadically available, such as the mesopelagic and deep ocean.

The co‐occurrence of sulphur oxidation genes alongside Calvin cycle‐related genes in the surface and subsurface layers suggests that thiosulfate may be utilised as an electron donor in microbial energy metabolism, driving sulphur oxidation processes as previously reported in the deep ocean (Srivastava et al. 2023). This metabolic strategy allows these microbes to simultaneously oxidise thiosulfate and fix carbon through the Calvin cycle, providing energy to sustain growth while avoiding competition with heterotrophs. Additionally, CO_2_ reduction/fixation genes, such as formyl‐methanofuran dehydrogenase subunit‐encoding genes (fwdA and fwdB), were more abundant in meso‐ and bathypelagic layers than in surface waters. This finding suggests that the reductive acetyl‐CoA pathway may function in tandem with methane metabolism among Euryarchaeota, such as *Methanosarcinales* (Figures 5a and 6) (Schöne et al. 2022). Finally, our data confirmed the global presence of phosphonate‐related genes throughout the entire water column (Lockwood et al. 2022), with a higher prevalence in the bathypelagic and surface ocean (Figure 7).

## 6 Conclusion

The microbial communities were well stratified across different depths, where different microbial groups occupy and thrive in distinct ecological niches based on their metabolic capabilities and resource requirements. The meso‐ and bathypelagic ocean layers are environments with limited resources, where different microbial strategies are employed to utilise pulsed and short‐term inputs of various energy sources and substrates. Both chemolithoautotrophy (where organisms obtain energy from inorganic chemicals) and heterotrophy (where organisms rely on organic compounds from other organisms) play significant roles. However, our findings also highlight the importance of mixed metabolic strategies where organisms utilise both inorganic and organic carbon sources. These findings highlight the importance of obtaining depth‐resolved profiles from a broader range of oceanic regions, including those with OMZs, to better understand spatial variability in microbial community structure and function and to uncover potentially novel metabolic processes.

Finally, our data showed that even when genetic sequences are classified into known taxonomic groups, their functions may still be unknown or poorly understood. Most of the genes found in our metagenomes were not taxonomically or functionally classified, indicating that key components of the ocean's prokaryotes remain unknown.

## Author Contributions

**Daniele De Corte:** conceptualization, investigation, validation, methodology, writing – review and editing, funding acquisition, writing – original draft, visualization, formal analysis. **Leon Dlugosch:** writing – review and editing, methodology, validation, visualization, data curation, formal analysis. **Abhishek Srivastava:** writing – review and editing, methodology, visualization, formal analysis. **Meinhard Simon:** writing – review and editing, funding acquisition. **Dennis A. Hansell:** writing – review and editing, funding acquisition. **Sarah Bercovici:** writing – review and editing, visualization, methodology. **Monica Orellana:** writing – review and editing, funding acquisition.

## Conflicts of Interest

The authors declare no conflicts of interest.

## Supporting information


**Figure S1:** Venn diagram of the prokaryotic community's core members across different depth layers (a). Taxonomic affiliation of the prokaryotic core members (b).

**Figure S2:** Heatmap of the detected genes (KOs) across the longitudinal transect and the entire water column.

**Figure S3:** Spearman correlation between indexes (diversity, richness and evenness indexes from mOTUS and KOs) and different physical–chemical and biological variables.

**Figure S4:** DOC, mOTUs and KOs diversity plots throughout the longitudinal transect in the whole depth profile.

## Data Availability

Sequence data are available at the NCBI Sequence Read Archive (SRA) under the BioProject accession number PRJNA1002880 (https://www.ncbi.nlm.nih.gov/bioproject/?term=PRJNA1002880). All data described are available upon request from the corresponding author.
