# Supplementary figures and images for: Taxonomic and Functional Features of Surface to Deep‐Sea Prokaryotic Communities in the Eastern North Pacific Ocean
Source: Environ Microbiol Rep. 2025 Aug 6;17(4):e70170. doi: 10.1111/1758-2229.70170 (PMC12328064; doi:10.1111/1758-2229.70170)

Figure.S1

a)

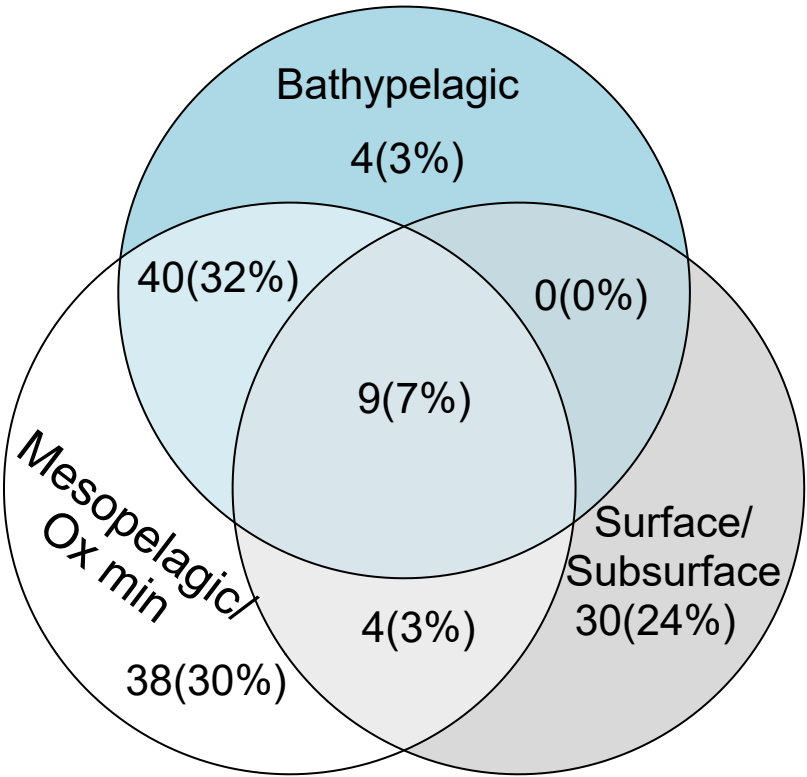

b)

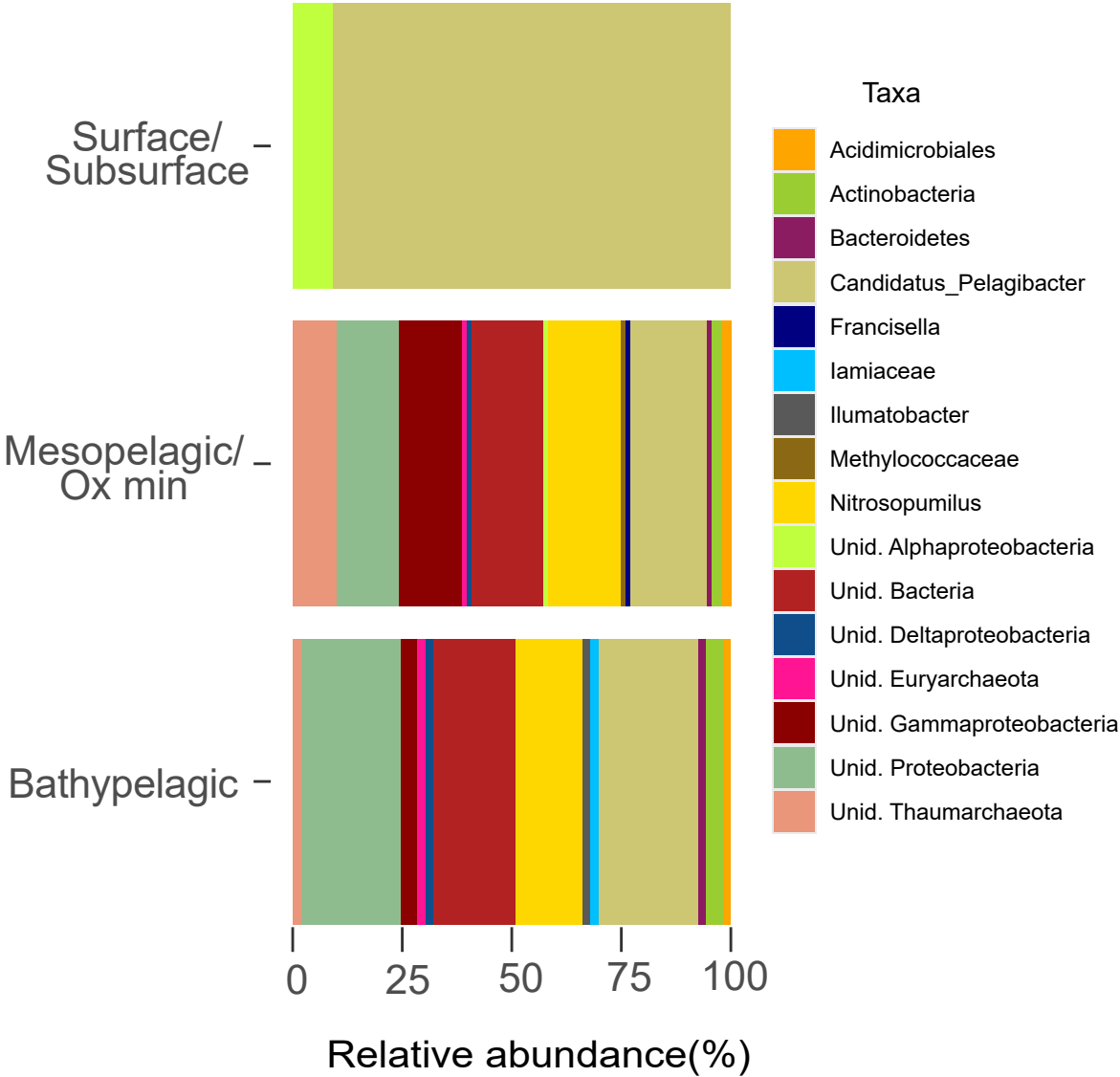

Supplement: Supplementary file 1 — Figure S1: Venn diagram of the prokaryotic community's core members across different depth layers (a). Taxonomic affiliation of the prokaryotic core members (b). [file EMI4-17-e70170-s004.pdf]

Figure.S2

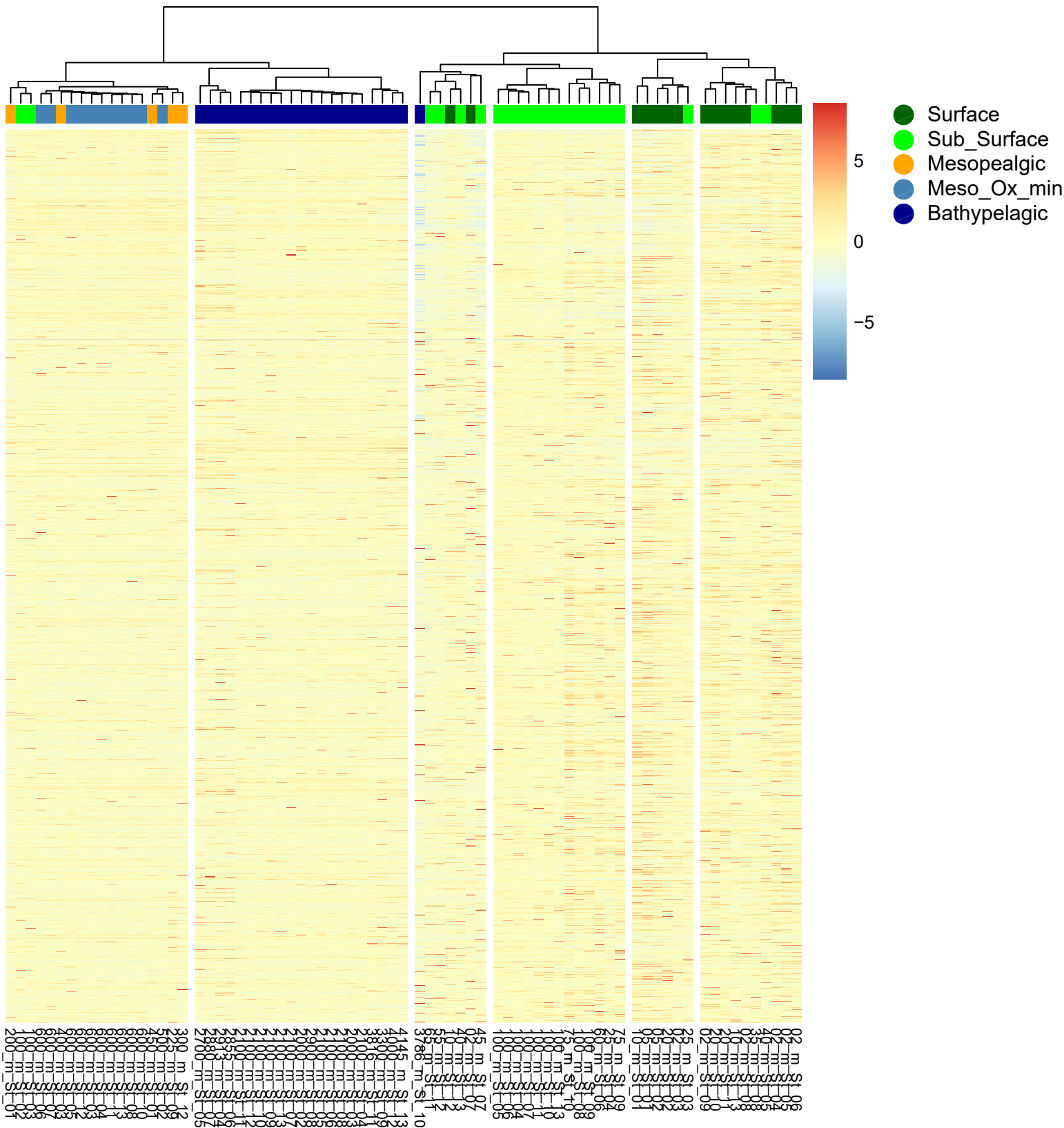

Supplement: Supplementary file 2 — Figure S2: Heatmap of the detected genes (KOs) across the longitudinal transect and the entire water column. [file EMI4-17-e70170-s001.pdf]

Figure.S3

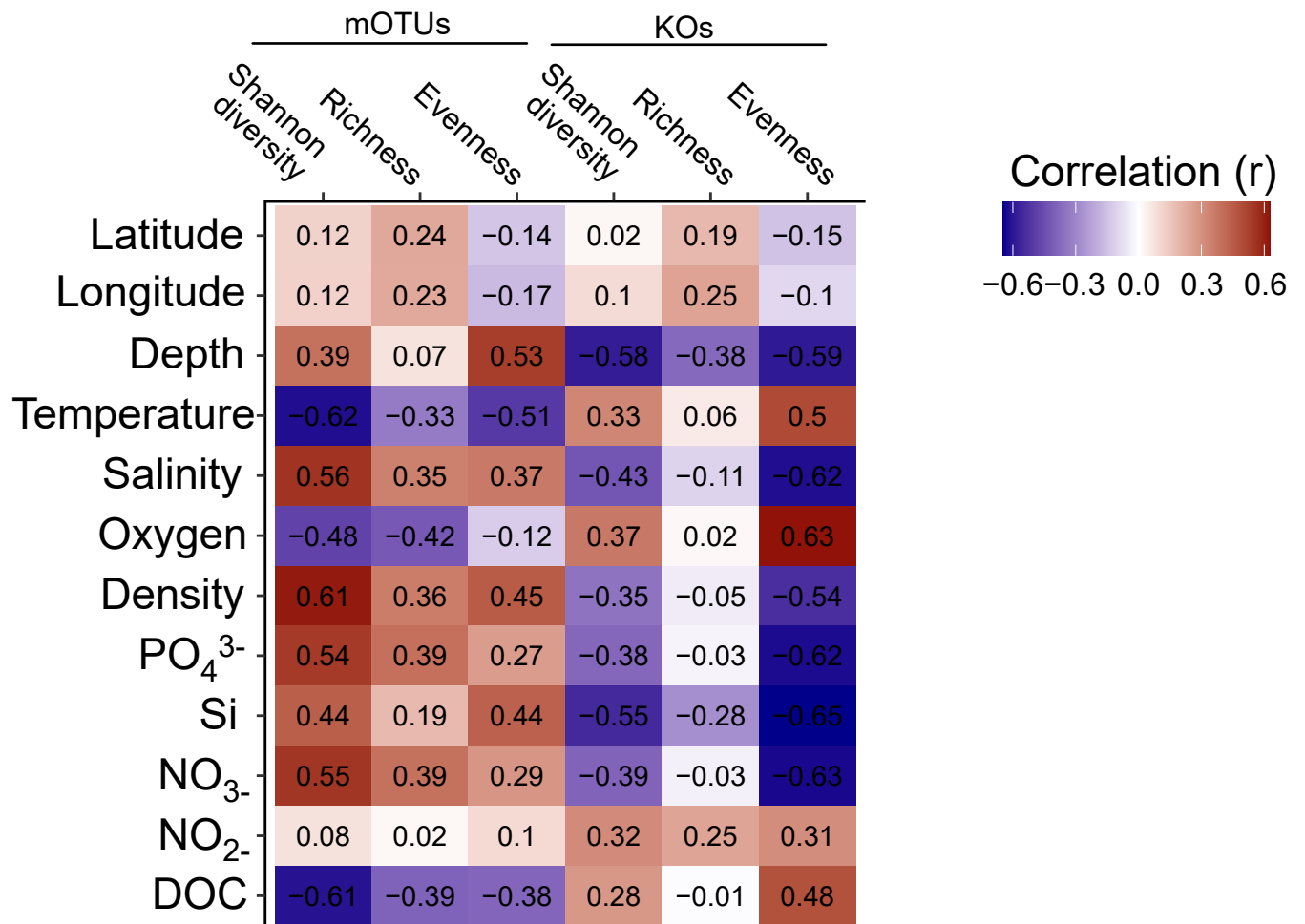

Supplement: Supplementary file 3 — Figure S3: Spearman correlation between indexes (diversity, richness and evenness indexes from mOTUS and KOs) and different physical–chemical and biological variables. [file EMI4-17-e70170-s003.pdf]

Figure.S4

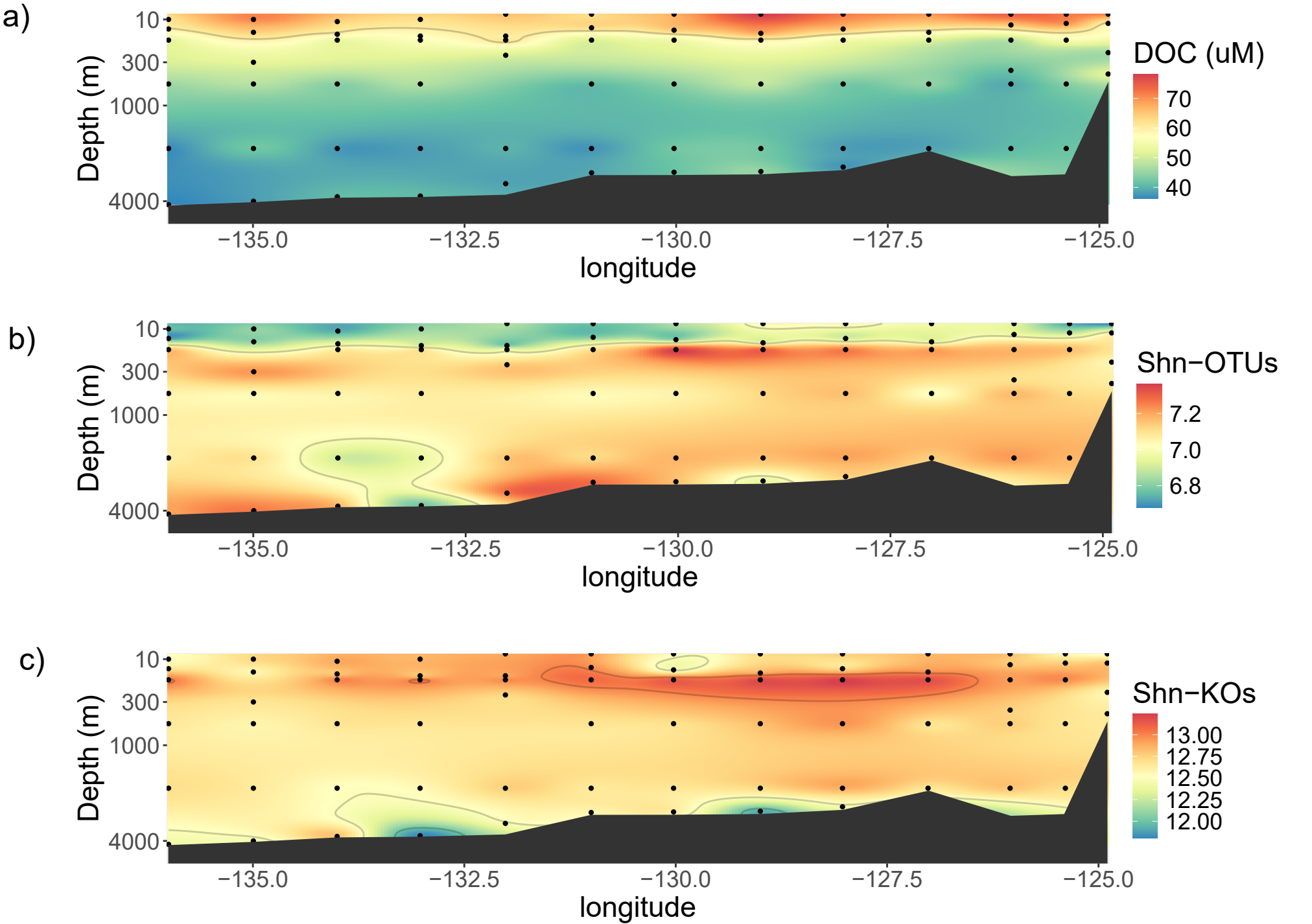

Supplement: Supplementary file 4 — Figure S4: DOC, mOTUs and KOs diversity plots throughout the longitudinal transect in the whole depth profile. [file EMI4-17-e70170-s002.pdf]
